# Predicting the HMA-LMA Status in Marine Sponges by Machine Learning

**DOI:** 10.3389/fmicb.2017.00752

**Published:** 2017-05-08

**Authors:** Lucas Moitinho-Silva, Georg Steinert, Shaun Nielsen, Cristiane C. P. Hardoim, Yu-Chen Wu, Grace P. McCormack, Susanna López-Legentil, Roman Marchant, Nicole Webster, Torsten Thomas, Ute Hentschel

**Affiliations:** ^1^Centre for Marine Bio-Innovation, University of New South WalesSydney, NSW, Australia; ^2^School of Biological, Earth and Environmental Sciences, University of New South WalesSydney, NSW, Australia; ^3^Laboratory of Microbiology, Wageningen UniversityWageningen, Netherlands; ^4^Departamento de Invertebrados, Museu Nacional, Universidade Federal do Rio de JaneiroRio de Janeiro, Brazil; ^5^RD3 Marine Microbiology, GEOMAR Helmholtz Centre for Ocean Research Kiel and Christian-Albrechts UniversityKiel, Germany; ^6^Zoology, Ryan Institute, School of Natural Sciences, National University of Ireland GalwayGalway, Ireland; ^7^Department of Biology and Marine Biology, and Center for Marine Science, University of North CarolinaWilmington, NC, USA; ^8^Centre for Translational Data Science, School of Information Technologies, University of SydneySydney, NSW, Australia; ^9^Australian Institute of Marine ScienceTownsville, QLD, Australia; ^10^Australian Centre for Ecogenomics, School of Chemistry and Molecular Biosciences, University of QueenslandSt. Lucia, QLD, Australia

**Keywords:** marine sponges, microbiome, 16S rRNA gene, microbial diversity, symbiosis, random forest

## Abstract

The dichotomy between high microbial abundance (HMA) and low microbial abundance (LMA) sponges has been observed in sponge-microbe symbiosis, although the extent of this pattern remains poorly unknown. We characterized the differences between the microbiomes of HMA (*n* = 19) and LMA (*n* = 17) sponges (575 specimens) present in the Sponge Microbiome Project. HMA sponges were associated with richer and more diverse microbiomes than LMA sponges, as indicated by the comparison of alpha diversity metrics. Microbial community structures differed between HMA and LMA sponges considering Operational Taxonomic Units (OTU) abundances and across microbial taxonomic levels, from phylum to species. The largest proportion of microbiome variation was explained by the host identity. Several phyla, classes, and OTUs were found differentially abundant in either group, which were considered “HMA indicators” and “LMA indicators.” Machine learning algorithms (classifiers) were trained to predict the HMA-LMA status of sponges. Among nine different classifiers, higher performances were achieved by Random Forest trained with phylum and class abundances. Random Forest with optimized parameters predicted the HMA-LMA status of additional 135 sponge species (1,232 specimens) without *a priori* knowledge. These sponges were grouped in four clusters, from which the largest two were composed of species consistently predicted as HMA (*n* = 44) and LMA (*n* = 74). In summary, our analyses shown distinct features of the microbial communities associated with HMA and LMA sponges. The prediction of the HMA-LMA status based on the microbiome profiles of sponges demonstrates the application of machine learning to explore patterns of host-associated microbial communities.

## Introduction

Sponges (Porifera) represent one of the oldest, still extant animal phyla. Fossil evidence dating back 600 million years ago shows their existence in the Precambrian (Yin et al., 2015) long before the radiation of all other animal phyla. Sponges are globally distributed in all aquatic habitats from warm tropical reefs to the cold deep sea and are even present in freshwater lakes and streams (van Soest et al., 2012). As sessile filter feeders, sponges are capable of pumping seawater at rates up to thousands of liters per kilogram of sponge per day (Vogel, 1977; Weisz et al., 2008). Small particles are retained from the incoming seawater and transferred into the mesohyl interior where they are digested by phagocytosis (Bell, 2008; Southwell et al., 2008; Maldonado et al., 2012).

Sponges are associated with microbial communities, with representatives of 41 different prokaryotic phyla thus far recovered from sponges, from which 13 phyla were shared among the 81 host species surveyed (Thomas et al., 2016). The sponge-associated microorganisms carry out functions related to nutrient cycling including carbon, nitrogen, and possibly sulfur and vitamin metabolism (Taylor et al., 2007; Bayer et al., 2008; Hentschel et al., 2012) as well as to secondary metabolism and chemical defense (Wilson et al., 2014). Sponge species were observed to harbor dense communities of symbiotic microorganisms in their tissues, while others were found essentially devoid of microorganisms (Reiswig, 1974). They were firstly termed “bacterial sponges” and “non-symbiont harboring, normal sponges” (Reiswig, 1981) and later the terms high microbial abundance” (HMA) and “low microbial abundance” (LMA) were used (Hentschel et al., 2003). Bacterial densities in HMA sponges are two to four orders of magnitude higher than in LMA sponges (Hentschel et al., 2006). In HMA sponges, microbial biomass can comprise up to one third of the total sponge biomass (Vacelet, 1975). HMA microbiomes are exceedingly complex, and LMA microbiomes are largely restricted to *Proteobacteria* as well as *Cyanobacteria* (Hentschel et al., 2006; Weisz et al., 2007; Kamke et al., 2010; Gloeckner et al., 2012; Schmitt et al., 2012; Giles et al., 2013). Functional gene content (Bayer et al., 2014), pumping rates (Weisz et al., 2008), and carbon and nitrogen compounds exchange (Ribes et al., 2012) were found to differ in respect to the HMA-LMA dichotomy. However, how the documented HMA-LMA status of sponges may impact the animal's physiology and metabolism as well as the surrounding environment is only beginning to be elucidated. The largest effort to characterize the HMA or LMA status of sponges thus far was performed by Gloeckner et al. (2014), who inspected 56 sponge species by transmission electron microscopy (TEM) and diamidino-2-phenylindole (DAPI) counting. Considering that more than 8,500 formally described sponge species exist and that the true diversity is still much higher (van Soest et al., 2012), a comprehensive survey of the HMA-LMA pattern would be a difficult and laborious undertaking.

Machine learning deals with the creation and evaluation of algorithms designed to recognize, classify, and predict patterns from existing data (Tarca et al., 2007). In supervised machine learning, the algorithms (classifiers) learn rules from features of labeled objects, known as training data, to infer the objects' labels (Sommer and Gerlich, 2013). Ultimately, these rules can be applied to predict the labels of unobserved objects. Supervised machine learning has been applied to predict biological features of different dimensions, ranging from molecular biology to macro ecology (Lawler et al., 2006; Petersen et al., 2011). Despite this, few publications have explored the power of machine learning to predict host characteristics based on microbiome patterns, such as the recent predictions made for human health and ethnicity (Mason et al., 2013; Walters et al., 2014). The present study was aimed to compare alpha and beta diversities between HMA and LMA sponge samples, to identify differently abundant prokaryotic taxa in HMA and LMA sponge species, and to predict the HMA-LMA status of sponges by machine learning. We demonstrate here that machine learning algorithms allow the accurate classification of the HMA-LMA status of marine sponges based only on the taxonomic profiles of samples' microbiomes.

## Materials and methods

### Data collection and determination of HMA-LMA status

Sponge-associated microbial community data were retrieved from the Sponge Microbiome Project dataset (Moitinho-Silva et al., in review). Briefly, sample processing and sequencing were performed by the Earth Microbiome Project (http://www.earthmicrobiome.org, Gilbert et al., 2014). Amplicon data analysis was conducted by Moitinho-Silva et al. (in review). The dataset consists of V4 hypervariable region of 16S rRNA gene sequences clustered at 97% similarity into Operational Taxonomic Units (OTU) and their taxonomic classification. In this study, samples annotated as diseased or as part of stress experiments were excluded, as were samples with <23,450 sequences, which corresponded to the first quartile of sequence counts per sample. Samples obtained from taxonomically identified sponge species with at least three replicates were used for the analyses. To account for difference in sequencing depth, the OTU abundance matrix was rarefied to 23,455 sequences per sample.

Classification of sponge species as either HMA or LMA was based on an electron microscopical survey (Gloeckner et al., 2014). Additionally, six species were classified in this study based on TEM. Altogether, 575 samples, representing 36 sponge species of known HMA-LMA status (*n* = 19 for HMA and *n* = 17 for LMA), were used for diversity and composition comparisons and as the machine learning training data (Supplementary Table 1). A total of 1232 samples, representing 135 sponge species of unknown HMA-LMA status, were then queried by machine learning approach (Supplementary Table 2). Samples ids are provided in Supplementary Table 3.

### Transmission electron microscopy (TEM)

Additional sponge samples were collected by SCUBA diving and processed for TEM by four different laboratories. *Ircinia variabilis* specimens (*n* = 3) were collected in March 2010, at 8–12 m depth at Mar Menuda (Tossa de Mar, Mediterranean Sea; 41°43′13.62″N, 2°56′26.90″E). *Petrosia ficiformis* specimens (*n* = 3) were collected in December 2011, at 8–11 m depth, at La Depuradora (L'Escala, Mediterranean Sea; 42°7′29″N, 3°7′57″E). These samples were processed for TEM as described in Erwin et al. (2012). *Rhopaloeides odorabile* specimens (*n* = 3) were collected in June 1999 from 8 m depth at Davies Reef (Northeast Australian Shelf—Great Barrier Reef; 18°50′33.48″S, 147°37′37.08″E). The samples were processed for TEM following Webster and Hill (2001). *Dysidea fragilis* and *Halichondria panicea* samples (*n* = 3) were collected at Coranroo (Co Clare, West Coast of Ireland, 53°8′29″N, 09°0′34″W) in 2012 and 2014, respectively. These were processed for TEM following Stephens et al. (2013). *Erylus formosus* specimens (*n* = 2) were collected from Bocas del Toro (Panama) in 2012. In the present study, the same *E. formosus* individuals were used for TEM analysis and for amplicon sequencing. After collection, samples were fixed in 4% paraformaldehyde followed by post-fixation in a 2% solution of osmium tetroxide in 0.1 M cacodylate buffer/11% sucrose. The samples were dehydrated in a graded ethanol series and embedded in LR White resin. Ultrathin sections were prepared with an ultramicrotome (Reichert Ultracut S, Leica, Austria). To obtain contrast, the sections were double stained with uranyl acetate replacement stain followed by lead citrate staining. TEM images were taken with a Tecnai G2 Spirit BioTwin TEM (80 kV, FEI, USA) at the Central Microscopy of University of Kiel (Germany).

### Experimental design used in diversity analyses

Alpha and beta diversities were compared between HMA and LMA samples (HMA-LMA status, fixed effect, 2 levels), taking into account the collection site (geographic region, random effect, 9 levels) and the sponge species (host identity, random effect, 36 levels). Samples were assigned into geographic regions based on their coordinates following Large Marine Ecosystems of the World definitions (http://www.lme.noaa.gov; Supplementary Table 1). The host identity factor was nested in the interaction between HMA-LMA status and geographic region.

### Statistical analysis of alpha diversity

Rarefaction curves were constructed to investigate the recovery of OTUs as a function of sequencing depth with mothur v. 1.37.6 (Schloss et al., 2009). Alpha diversity indices were obtained from the OTU abundance matrix. OTU counts, the Chao, and ACE estimators (O'Hara, 2005; Chiu et al., 2014) were considered indicators of community richness. Inverted Simpson (InvSimpson), Shannon, and Pielou's evenness indices were considered as indicators of community diversity. Calculation of alpha diversity indices was performed with the R package vegan v. 2.3-5 (Oksanen et al., 2016). The effect of HMA-LMA status on the alpha diversity was examined using likelihood ratio tests that compared two linear models with mixed effects: one with the HMA-LMA factor, i.e., the full model, and another without the HMA-LMA factor, i.e., the null model. For this purpose, linear mixed models were fitted by maximum likelihood with the function *lmer* of the R package lme4 (Bates et al., 2015) with the parameter REML set to false. Cell mean parameterization and confidence intervals were obtained from the full model. For each model, residuals vs. fits and normal quantile plots were inspected to verify that assumptions of normality, constant variance, and linear relationship were kept. *P*-values were calculated with ANOVA function based on χ^2^ statistic with an alpha level of 5%.

### Statistical analysis of beta diversity

Distance-based multivariate analysis of the microbial communities was carried out at the OTU level as well as the taxonomic levels of phylum, class, order, family, genus, and species. For each taxonomic level, OTU abundances were grouped according to Greengenes classification. Inferences on community structure were based on Bray–Curtis dissimilarities of samples. Patterns between microbial community structures of sponge samples were inspected using Non-metric Multidimensional Scaling (NMDS) performed with the vegan package. The effects of factors in the described experimental design were tested with PERMANOVA (Anderson, 2001), using square root transformed data and type III sum of squares. Estimates of components of variation were calculated in PERMANOVA. Because differences between groups in PERMANOVA could be due to location, dispersion, as well as location and dispersion (Anderson et al., 2008); homogeneity of multivariate dispersions was examined with PERMDISP (Anderson, 2006). *P*-values of PERMANOVA and PERMDISP were calculated using 999 permutations. Distance-based multivariate statistics were performed with PERMANOVA v. 1.0.1 implemented in PRIMER v. 6.1.11 (PRIMER-E, UK) with an alpha level of 5%.

### Statistical analysis of taxa abundances in HMA and LMA species

The detection of microbial taxa that were differentially abundant between HMA and LMA sponges was conducted at the host species level because analysis of microbial diversities indicated that this factor was responsible for a large part of the variation observed (see Table 1 and Supplementary Table 4). Therefore, microbial abundances of samples in phylum, class, and OTU abundance matrices were averaged by sponge species. Generalized linear model was separately fitted to each taxon using negative binomial distribution with the R package Mvabund (Wang et al., 2012), given a mean-variance relationship was observed. Univariate log-likelihood ratio statistic and *P*-value were calculated after 999 bootstraps. Mean and confidence interval estimates are presented in percentages for taxa with significant effects (alpha level of 5%).

**Table 1:** **The effect of HMA-LMA status, geographic region, and host identity on microbial communities based on OTU abundances**.

**Source**	***df***	**MS**	**Pseudo-*F***	**P (perm)**	Var comp^**^
HMA-LMA status	1	41,744	5.5289	0.002	34.238
Geographic region	8	15,266	1.4992	0.012	13.223
HMA-LMA status × geographic region^*^	5	15,398	1.6644	0.003	17.741
Host identity (HMA-LMA status × geographic region)	37	20,925	12.249	0.001	41.229
Residual	523	1708.3			41.332

*PERMANOVA analysis was performed with Bray–Curtis dissimilarities between samples obtained from square root transformed OTU abundances*.

* *Term has one or more empty cells*.

** *Estimates of components of variation are shown in squared units of Bray–Curtis dissimilarity*.

### Prediction of HMA-LMA status by machine learning

The capability of machine learning algorithms to classify unknown species into HMA or LMA sponges was evaluated. The training dataset was built on microbial community features from sponge samples of known HMA-LMA status (in Supplementary Table 1). Because specific sets of features can impact the accuracy of the classification, prediction performance was evaluated using the phylum, class, and OTU abundance matrices. Classifiers and their default parameters (listed in Supplementary Table 7) were chosen based on their availability on the Scikit-learn python package v. 0.17.1 (Pedregosa et al., 2011). The performance of each classifier was evaluated using a Leave One Out (LOO) per sponge species fashion, i.e., for each species, its samples were left out of the training set and the classifier was trained based on the remaining samples of other species. According to this procedure, each classifier predicted the HMA-LMA status of species for which samples were not present in the training set. The performance score was measured as the percentage of correctly classified samples. Further, parameter tuning was conducted on the classifier and datasets that presented the best performance, which were the Random Forest classifier and the abundance matrices on the phylum and class levels.

Random Forest is a non-parametric machine learning method consisting of a collection of tree-structured classifiers (Breiman, 2001; Chen and Ishwaran, 2012). Each tree is grown on replicates of the training set obtained by sampling. Here, we used bootstrapping as the sampling with replacement method. The result of Random Forest was obtained by averaging the probabilistic prediction of the classifiers as implemented in Scikit-learn (Pedregosa et al., 2011). The effect of different numbers of trees in the forest (ranging from 10 to 100) on the performance of Random Forest was compared. The maximum depth of the tree was set to “None” and features when looking for the best split was set to “auto.” Random Forest classification that presented higher performance was achieved with 50 trees in the forest. This parameter was used to predict the HMA-LMA status of sponge species without microscopical classification, i.e., unlabeled (Supplementary Table 2).

Classification results were summarized as percentage of species samples classified as HMA, where the values ranged from 0 to 100%. The proportion of samples classified as LMA was deduced from this percentage. Results obtained from phylum and class abundance matrices were clustered by affinity propagation (AP). AP clusters data points based on subsets of representative examples, which are identified among all data points (Frey and Dueck, 2007). Pairwise similarity was measured as negative squared Euclidean distances (Frey and Dueck, 2007). Exemplary preferences were set to the median, which is expected to result in a moderate number of clusters in comparison to a small number of clusters that result when the exemplary preferences are set to their minimum. Clusters were joined by exemplar-based agglomerative clustering (Bodenhofer et al., 2011). AP and exemplar-based agglomerative clustering were conducted using the R package apcluster v. 1.4.3 (Bodenhofer et al., 2011).

## Results

### HMA-LMA classification based on electron microscopy

**Figure 1:**
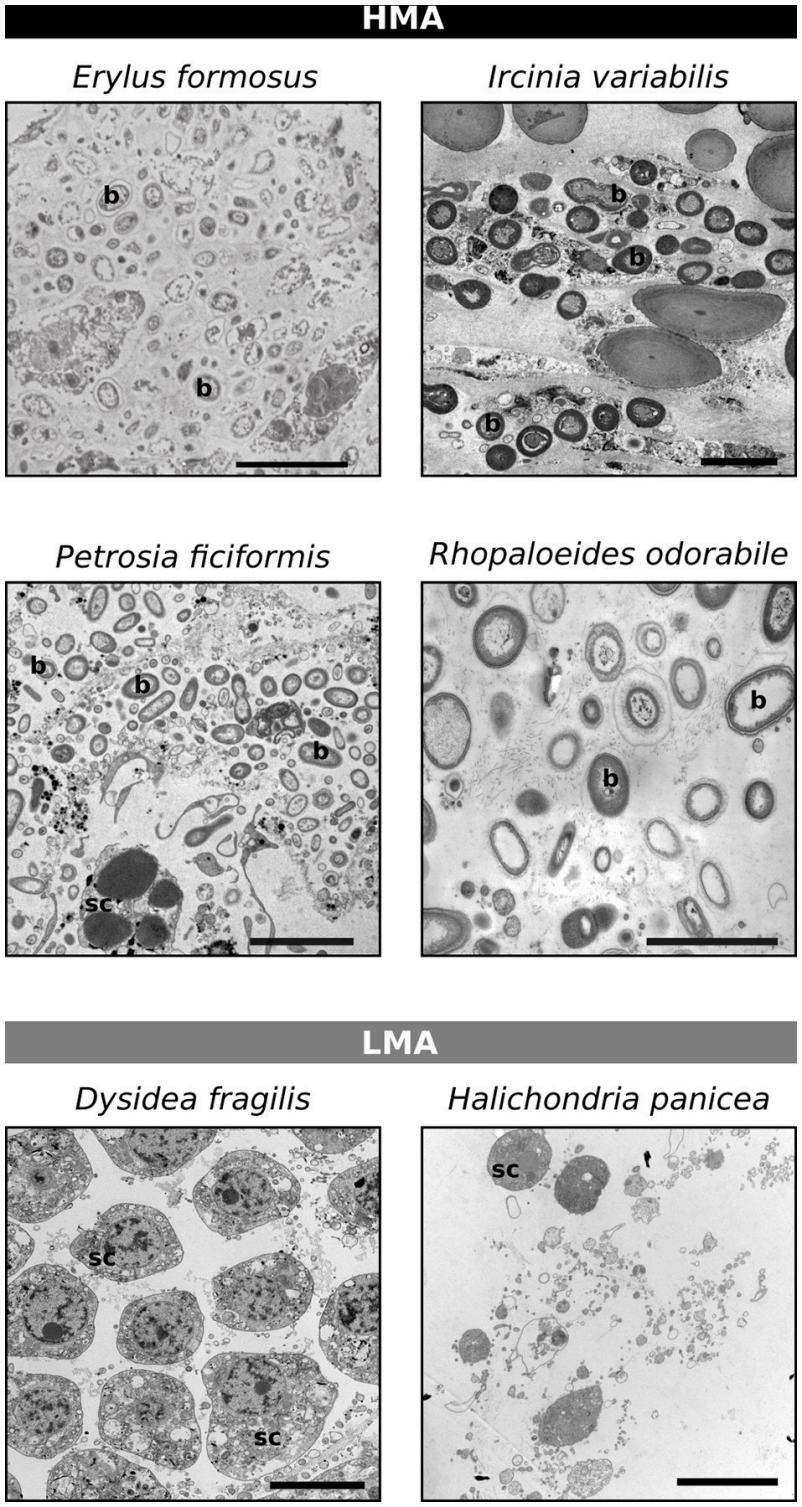
**Classification of the HMA-LMA status of sponges based on transmission electron microscopy**. Scale bars represent 5 μm, but vary in length. b, bacteria; sc, sponge cell.

Based on TEM observations, we report the HMA-LMA status of five additional sponge species that were not covered by Gloeckner et al. (2014). *I. variabilis, P. ficiformis*, and *R. odorabile* were classified as HMA sponges due to the presence of abundant and morphologically distinct microbial cells in the mesohyl (Figure 1). *I. variabilis* and *P. ficiformis* exhibited particularly high density of microbial cells in their mesohyl. The sponge species *D. fragilis* and *H. panicea* were classified as LMA because their mesohyl were largely devoid of microbial cells. In addition, the contradictory classification of *E. formosus* as being LMA (Gloeckner et al., 2014) or HMA (Easson and Thacker, 2014) was revisited and based on the present TEM images documenting large amounts of microorganisms (Figure 1), *E. formosus* was clearly identified as an HMA sponge.

### Alpha and beta diversities in HMA and LMA sponges

**Figure 2:**
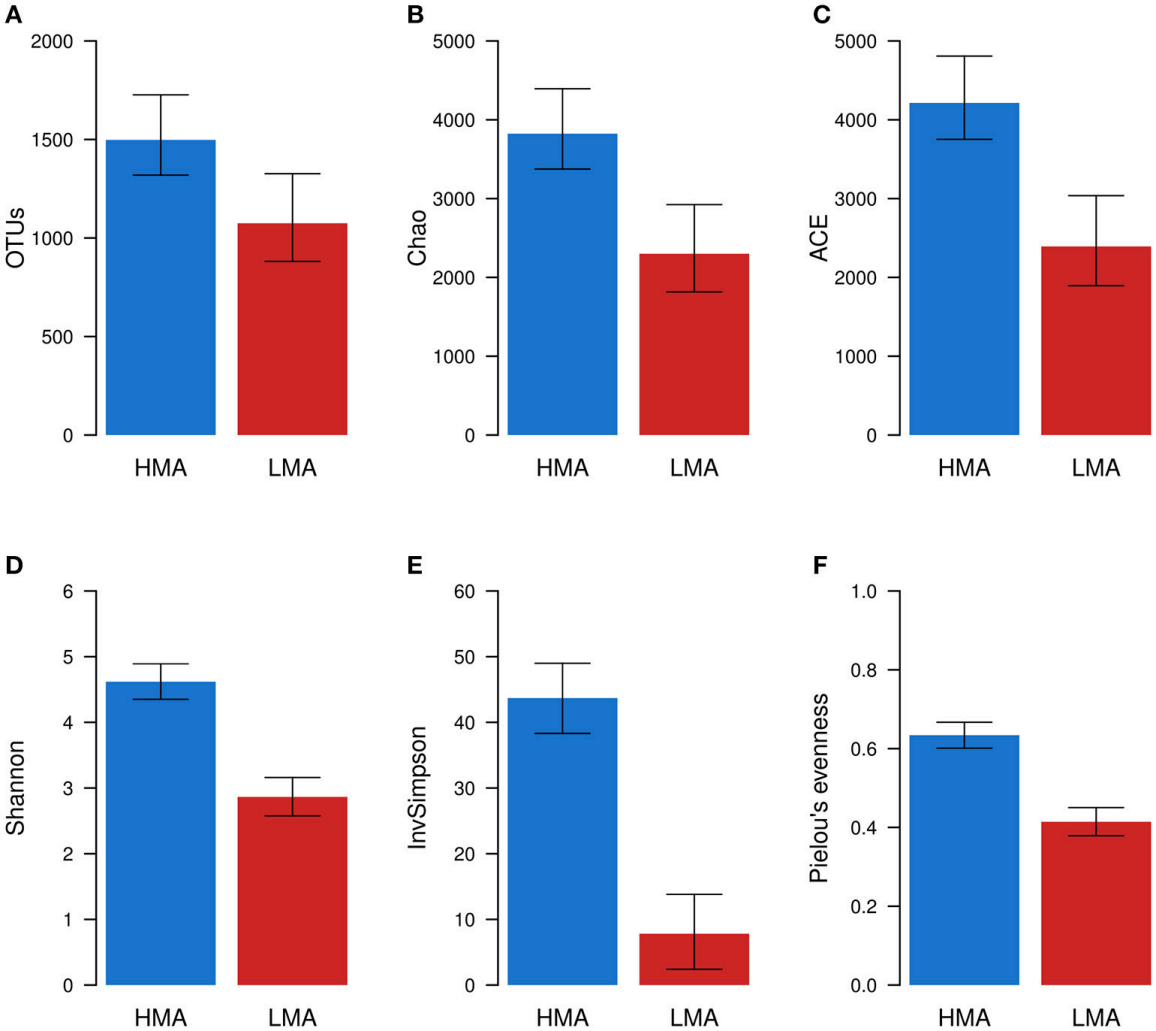
**Alpha diversity of HMA and LMA sponge samples**. Richness **(A–C)** and diversity **(D–F)** metrics were calculated for each sample (*n* = 575) using rarefied OTU abundances. Estimated mean and 95% confidence intervals were obtained from linear mixed models of alpha diversity metrics. The effect of HMA-LMA status was tested with Likelihood ratio tests. In this procedure, two linear models with mixed effects were compared, the full model and the null model. All metrics were significantly greater (ANOVA, *P* ≤ 0.001) in the HMA than in the LMA group.

Rarefaction curves indicated a broad gradient of sampling depths and number of OTUs (16S rRNA gene sequences clustered at 97% similarity) obtained for HMA and LMA samples (Supplementary Figure 1). When rarefaction curves were constructed based on the OTU table rarefied to 23,455 sequences per sample, the curves were more heterogeneous in LMA than HMA sponge samples. Alpha diversity measures were calculated from OTU abundances of sponge samples. All metrics were statistically significantly greater (ANOVA, *P* ≤ 0.001, Supplementary Table 4) in the HMA than in the LMA group. The HMA microbiomes were 1.4x–1.8x richer than the LMA microbiomes as measured by OTU counts and the estimators Chao and ACE (Figures 2A–C). The diversity of HMA microbiomes was 1.5x and 1.6x greater than LMA microbiomes according Shannon and Pielou's evenness measures respectively, while being 5.6x greater according to Inverted Simpson index (InvSimpson; Figures 2D–F).

**Figure 3:**
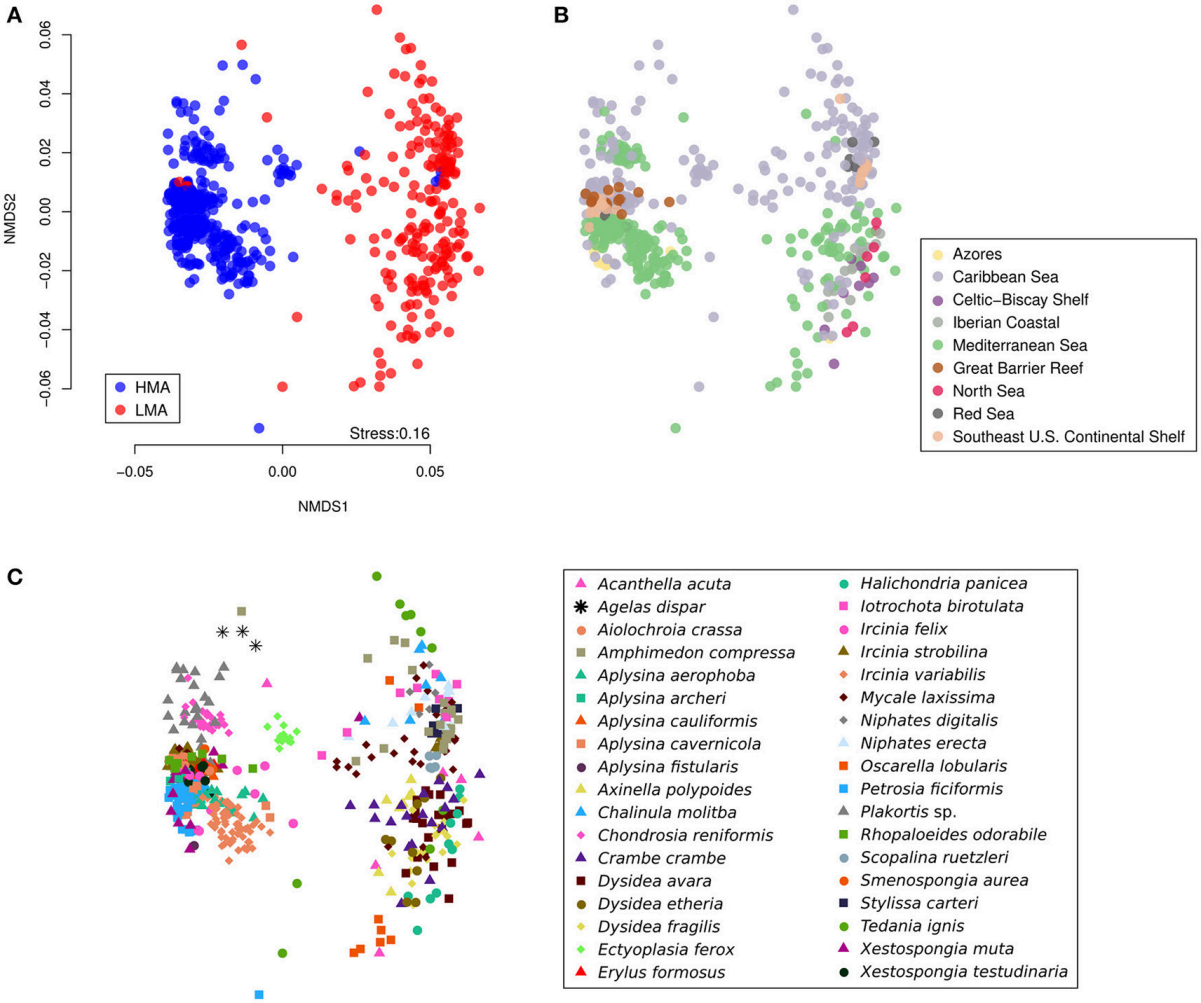
**Beta diversity of microbial communities associated with HMA and LMA sponge samples**. NMDS was conducted from Bray–Curtis dissimilarities between samples based on OTU abundances. The three displayed plots represent the same analysis, where sample symbols and colors stand for **(A)** HMA-LMA status, **(B)** geographic region, and **(C)** host identity.

A clear separation between the structures of microbial communities in HMA and LMA samples was observed in the Non-metric Multidimensional Scaling (NMDS) plot (Figure 3). A significant proportion of variation in the community structures [PERMANOVA, pseudo-*F*_(1, 523)_ = 5.5, *P* = 0.002] was explained by the HMA-LMA status, while controlling for the effects of geographic region, the interaction of region and HMA-LMA status, and the host identity (Table 1). As suggested by the NMDS plots, at least part of the observed differences between HMA and LMA samples was due to the difference in dispersion between groups, where HMA samples were less dispersed than LMA samples according to PERMDISP test (*t* = 14.9, *P* = 0.001, Supplementary Figure 2). A significant effect of geographic region on microbial community structure was observed [pseudo-*F*_(8, 523)_ = 1.5, *P* = 0.012], as well as of the interaction between HMA-LMA status and geographic region [pseudo-*F*_(5, 523)_ = 1.7, *P* = 0.003], and host identity [pseudo-*F*_(37, 523)_ = 12.2, *P* = 0.001; Table 1]. It is noteworthy, that the host identity explained most of the variation, followed by the HMA-LMA status, the interaction between geographic region and HMA-LMA status, and, lastly, geographic region alone (Table 1).

The effect of HMA-LMA status was observed when OTU abundances were grouped by microbial taxonomic levels (Table 2). This result is particularly remarkable considering the increasing number of sequences that cannot be assigned to a given taxon when deeper classification levels are considered. For instance, 5.75% of sequences were grouped as unclassified at phylum level and 98.24% were grouped as unclassified at the species level.

**Table 2:** **The effect of HMA-LMA status on microbial communities at different taxonomic ranks**.

**Level**	**MS**	**Pseudo-*F***	**P (perm)**	Unclassified sequences^*^
Phylum	21,816	12.172	0.001	5.75
Class	25,402	10.755	0.001	20.27
Order	184,850	8.7579	0.001	54.64
Family	31,065	10.029	0.001	64.11
Genus	33,377	9.9077	0.001	89.34
Species	33,061	9.6671	0.001	98.24
df:1, Res:224, Total:250				

*PERMANOVA analysis was performed with Bray–Curtis dissimilarities between samples obtained from square root transformed abundances. See Table 1 for full model*.

* *Percentage of sequences that fell in “unclassified” taxon during the taxonomic grouping of OTU abundances*.

### Identification of HMA and LMA indicator taxa

**Figure 4:**
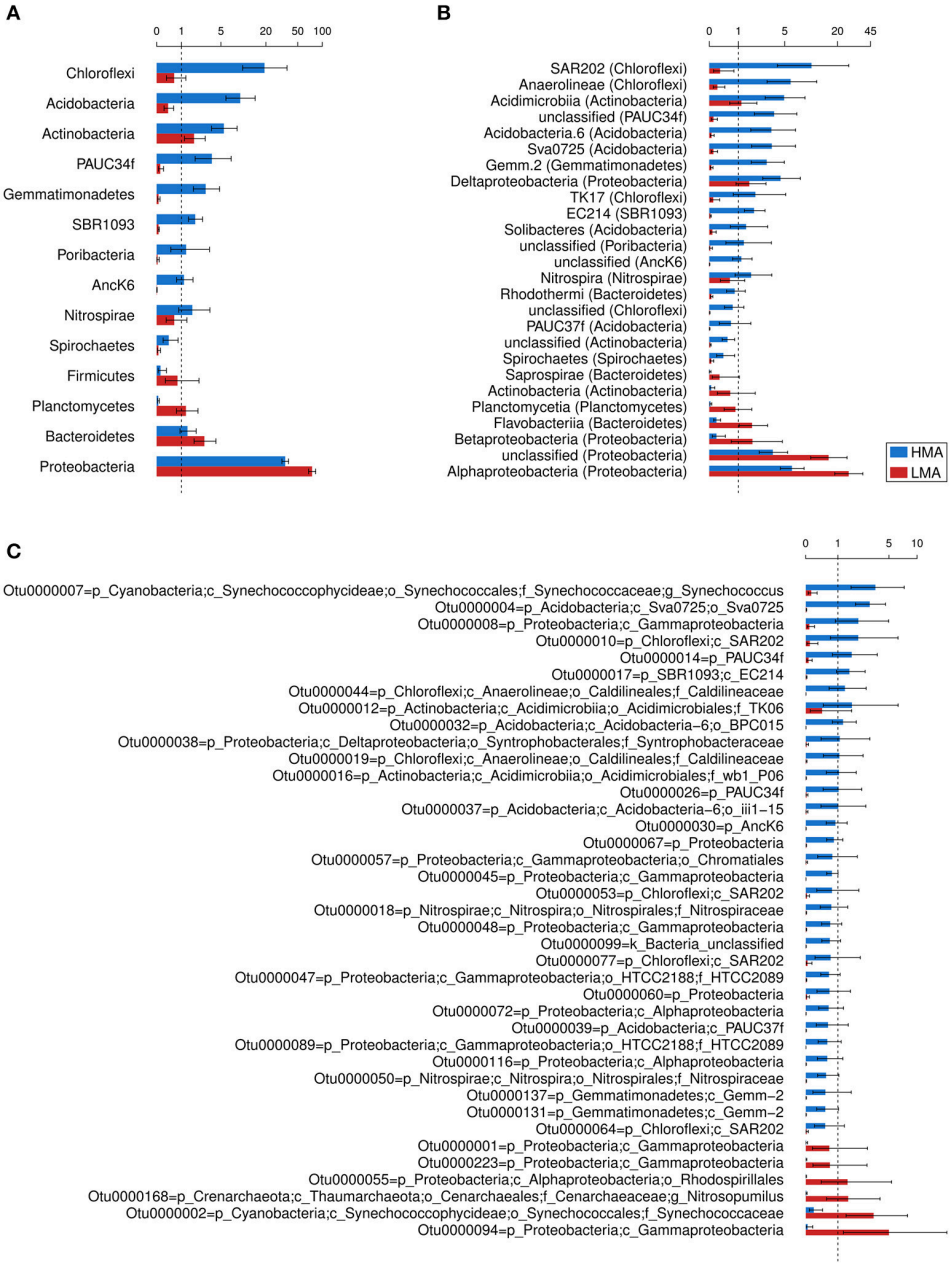
**Selection of differentially abundant bacterial and archaeal taxa in the microbiomes of HMA and LMA sponge species**. Estimated mean and 95% confidence intervals were obtained from negative binominal generalized linear models (HMA = 19, LMA = 17) and converted to percentages. **(A)** Phyla and **(B)** classes that differed in more than 0.25% of their mean relative abundance per group are displayed. **(C)** The cut-off for OTUs was 0.5% difference. The shown taxa resulted in *P* < 0.05. Classification of OTUs is shown down to their deepest taxonomic level.

The taxa that differed in abundance between HMA and LMA sponges (*P* < 0.05) were inspected with phylum, class, and OTU datasets (Figure 4). Other taxonomic levels were not included due to the large proportion of sequences assigned to unclassified taxa (>50%). On the phylum level, 14 phyla were significantly more abundant in HMA and 19 were more abundant in LMA species (Supplementary Table 5). Because these numbers included many low abundance phyla, we further considered only those that differed on average >0.25%. Accordingly, the phyla *Chloroflexi, Acidobacteria*, and *Actinobacteria* were more abundant in HMA sponges, followed by PAUC34f, *Gemmatimonadetes*, BR1093, *Poribacteria*, AncK6, *Nitrospirae*, and *Spirochaetes* (Figure 4A). The phyla *Proteobacteria, Bacteroidetes, Planctomycetes*, and *Firmicutes* were more abundant in LMA sponges. The classes SAR202, *Anaerolineae*, and *Acidimicrobiia* were more abundant in HMA sponges, followed by PAUC34f unclassified at class level, Acidobacteria.6, Sva0725, Gemm.2, *Deltaproteobacteria*, and others (Figure 4B and Supplementary Table 5). The classes *Alphaproteobacteria, Betaproteobacteria, Flavobacteriia, Planctomycetia, Actinobacteria*, and *Saprospirae* were more abundant in LMA sponges (Figure 4B). Microbiomes of LMA sponges were enriched in *Proteobacteria* unclassified at the class level over HMA microbiomes. A total of 2,322 OTUs were found to be differentially abundant between HMA and LMA groups (Supplementary Table 6). The taxonomic classification of the most abundant OTUs enriched in either HMA or LMA sponges corresponded to the results obtained for phylum and class level (Figure 4C). The two abundant OTUs assigned to the family *Synechococcaceae* represent a remarkable exception to the above pattern. Despite the fact that neither the phylum *Cyanobacteria* nor the class *Synechococcophycideae* was enriched in either group, the cyanobacterial Otu0000007 was more abundant in HMA sponges, while the cyanobacterial Otu0000002 was more abundant in LMA sponges. Similarly, despite the fact that *Thaumarchaeota* was not enriched in either of the groups, the thaumarchaeal Otu0000168 was more abundant in the LMA sponges. The large confidence intervals observed for some OTUs' means indicate that these OTUs are not evenly distributed among the sponge species within HMA or LMA groups. For example, Otu0000094, which had a mean in LMA sponges of 5.0% (95% confidence interval: 1.3%, 19.9%), were found in only 4 of the 17 LMA species with most of its sequences (19,738 out of 19,769 total) recovered from *Iotrochota birotulata*.

### Prediction of the HMA-LMA status by machine learning

**Figure 5:**
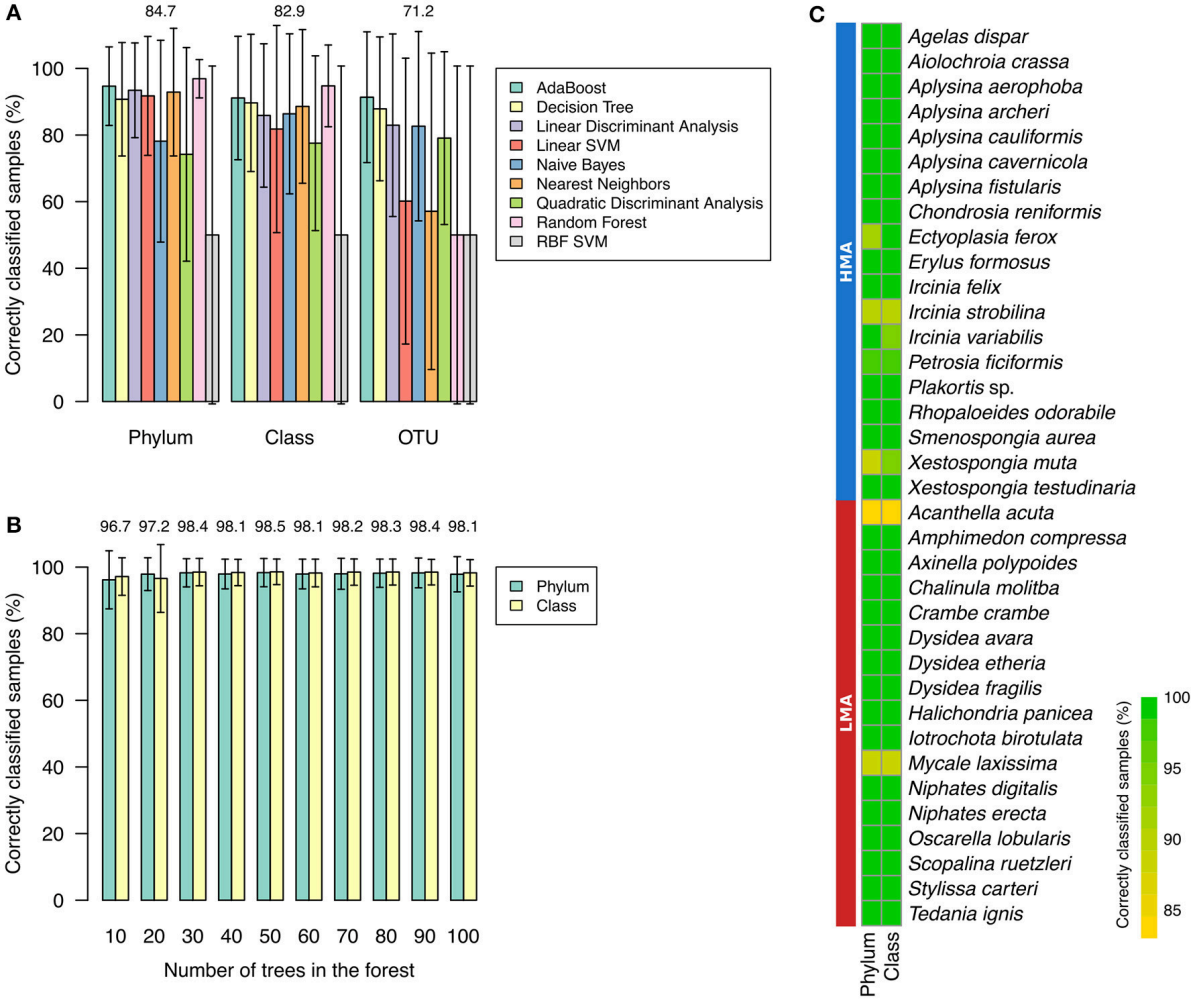
**Selection and standardization of classifiers. (A)** Performance of classifiers training on phylum, class, and OTU abundances. Percentage of correctly classified samples per species were averaged according to training tables. Weighted means were used due to the difference in number of HMA (*n* = 19) and LMA (*n* = 17) sponges. Error bars represent weighted standard deviations. **(B)** Performance of Random Forest for phylum and class datasets according to number of trees in the forest. Mean of weighted averages are displayed at the top of bars. **(C)** Performance of Random Forest (number of trees in the forest = 50) on classification of known HMA and LMA sponge species. Radial Basis Function kernel (RBF) and Support Vector Machine (SVM) are abbreviated.

To select the supervised machine learning algorithm (classifier) that was most appropriate to the task of predicting the HMA-LMA status, the performance of several classifiers were compared. Random Forest resulted in higher weighted means of correctly classified samples per species when training and validation was carried out with phylum (96.90% ± 5.75, weighted mean ± weighted standard deviation) and class abundances (94.75% ± 12.27; Supplementary Table 7, Figure 5A). On the other hand, AdaBoost performed better with OTU abundances (91.35% ± 19.63). Although AdaBoost performance was also high for phylum and class datasets (>91% weighted mean), it resulted in higher weighted standard errors, when compared to Random Forest (Supplementary Table 7). Thus, Random Forest was preferred over AdaBoost. Due to the overall low predictive value obtained for OTU abundance information (mean performance of 71.2%) in comparison to phylum (84.7%) and class (82.9%), we decided to perform downstream analysis based on the latter two datasets. The number of trees in the forest was further optimized for the Random Forest classifier. Highest overall performance, i.e., mean of performance for phylum and class datasets, was obtained for 50 trees in the forest (Figure 5B). Optimized Random Forest performance on phylum and class datasets resulted, respectively, in 98.3% ± 4.2 and 98.6% ± 3.8 of correctly classified samples clearly demonstrating that most samples for all species were correctly classified (Figure 5C).

**Figure 6:**
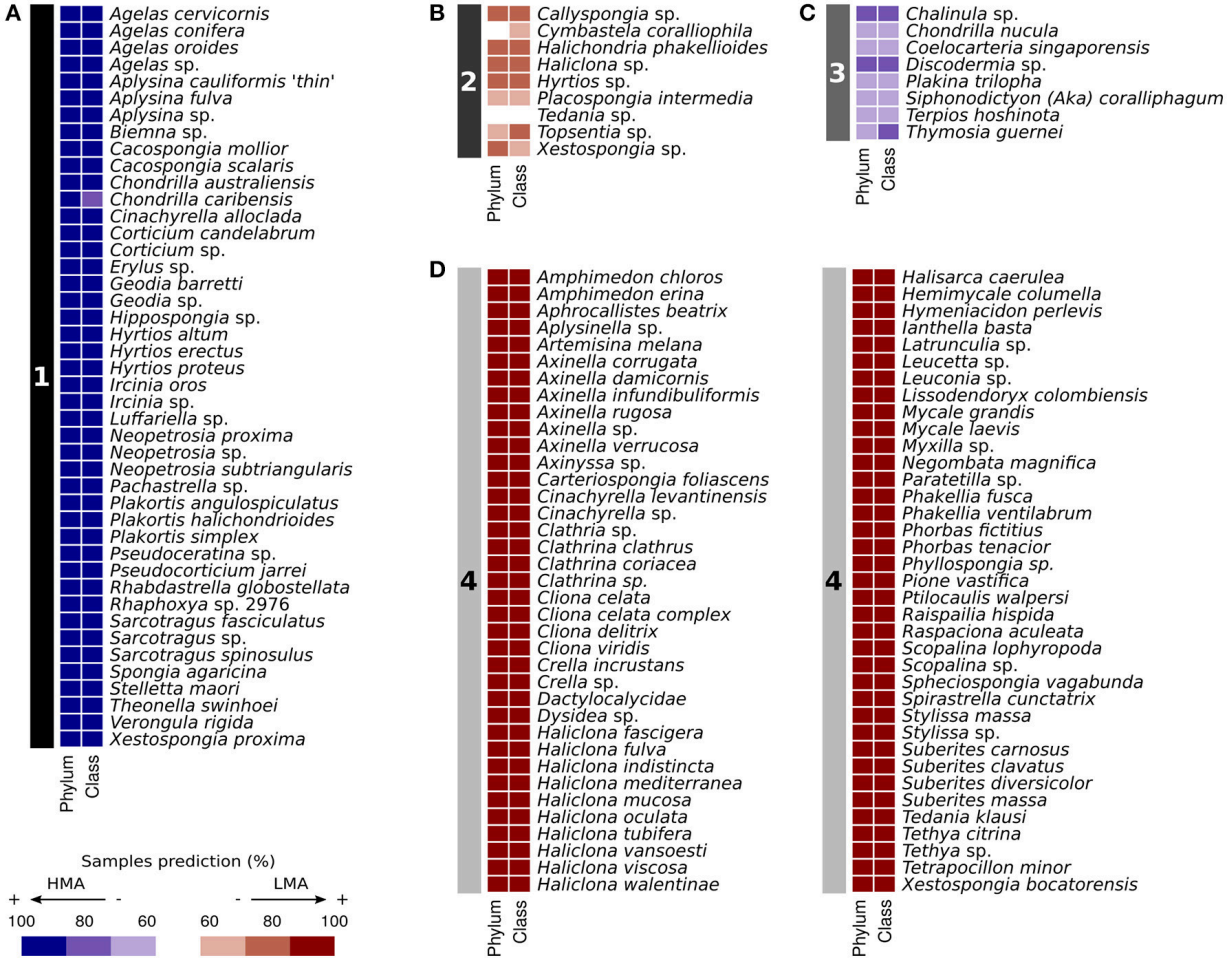
**Random Forest predictions of HMA-LMA status of previously uncharacterized sponge species (***n*** = 135)**. Prediction of samples were carried out by Random Forest (number of trees in the forest = 50) based on phylum and class abundances. Clustering of the classifier results (left numbered panel, **A–D**) were performed with affinity propagation. Color scheme of right panels represents percentage of samples predicted as either HMA or LMA.

Prediction of the HMA-LMA status of 135 sponge species without *a priori* knowledge was performed using Random Forest with optimized parameters. Four clusters were obtained by affinity propagation based on prediction results on phylum and class abundance information (Figure 6). Cluster 1 contained 44 species that were largely classified as HMA (84–100% of samples). Cluster 2 contained 9 species that were inconsistently classified as LMA (55–84% of samples). This included *Tedania* sp., for which 56% of samples were classified as LMA. Cluster 3 contained 8 species that were inconsistently classified as HMA (59–83% of samples). Cluster 4 grouped 74 species that were largely classified as LMA (86–100% of samples). Cluster 1 was grouped together with Cluster 3, while Cluster 2 was paired with Cluster 4 by exemplar-based agglomerative clustering (Supplementary Figure 3).

**Figure 7:**
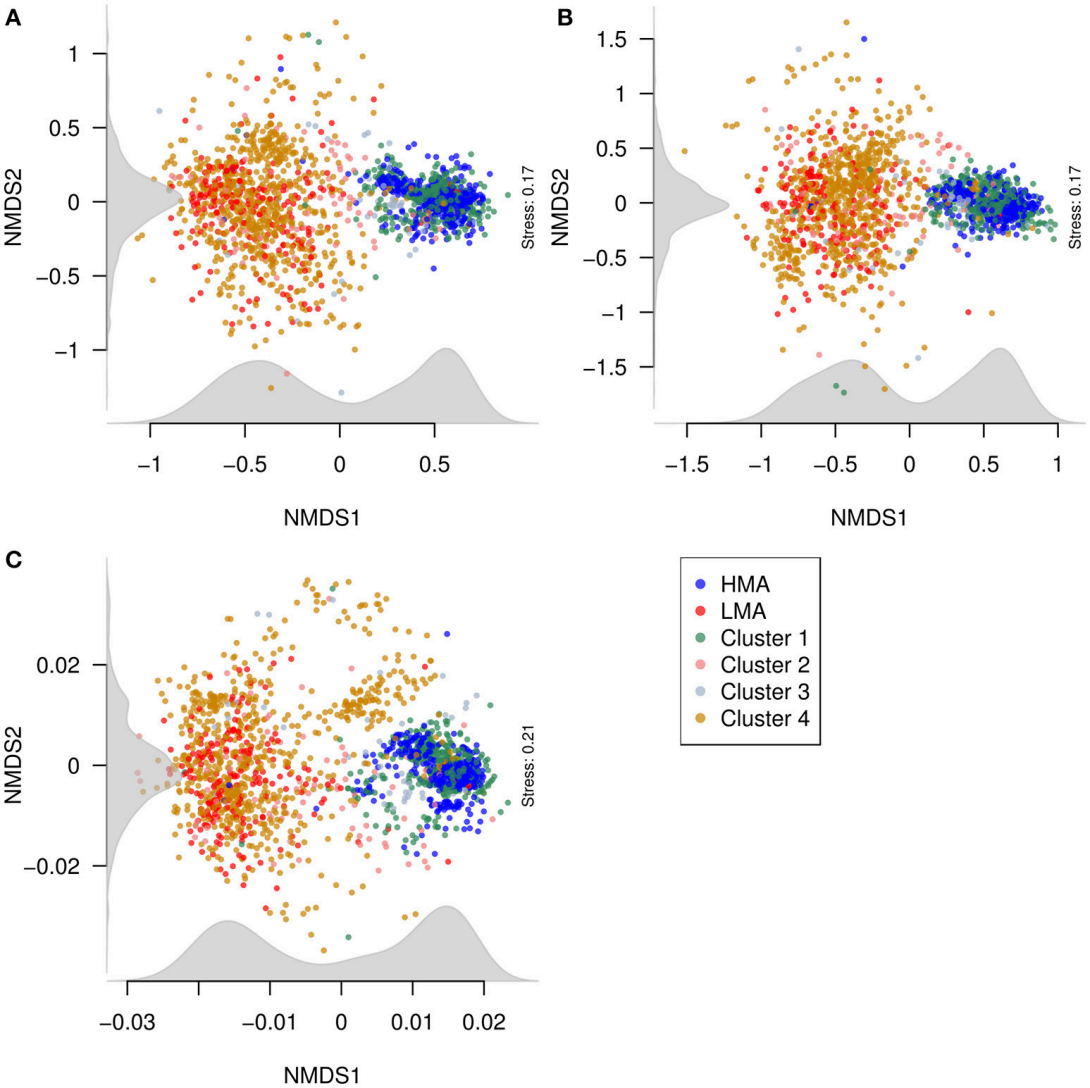
**Relationship between the structures of microbial communities (beta diversity) from classified and predicted sponges**. NMDS plots were constructed from Bray–Curtis dissimilarities between samples obtained from **(A)** phylum, **(B)** class, and **(C)** OTU abundances. Points correspond to samples and are colored according to the HMA-LMA classification and to the clusters obtained from Random Forest prediction results (see Figure 6). Density of points along the NMDS dimensions (axes) was plotted in gray.

To visualize the relationship between the structures of microbial communities from classified and predicted sponges, NMDS was conducted with the phylum, class, and OTU abundance matrices (Figure 7). Generally, samples from species in Clusters 1 and 3 closely localized with samples of HMA sponges. Likewise, samples from species in Clusters 2 and 4 closely localized with samples from LMA sponges. The distinction between the groups was clearer in the NMDS plots produced from the phylum and class datasets than in the plot produced from the OTU dataset. Nevertheless, NMDS plots from all three datasets suggest a bimodal pattern of structures of microbial communities in sponges, as displayed by the density of samples along the first NMDS dimension (x axis).

## Discussion

### Microbial diversity in HMA and LMA sponges

Studies that have characterized sponge microbial diversity in the context of the HMA-LMA dichotomy have so far been restricted to a handful of samples and/or species (e.g., Blanquer et al., 2013; Giles et al., 2013). In surveys that incorporated a larger number of species, the HMA-LMA dichotomy was only a minor part of the investigation (Schmitt et al., 2012; Easson and Thacker, 2014). Here, the microbiomes of 575 samples representing 36 species of known HMA-LMA status were characterized and statistically analyzed. The HMA-LMA status was predicted by machine learning for another 118 of 135 sponge species with high confidence (representing 1,094 samples). This effort represents the largest investigation of the HMA-LMA dichotomy so far and was possible due to the recent release of the Sponge Microbiome Project dataset (Moitinho-Silva et al., in review).

All alpha diversity metrics considered in this study showed that HMA sponges are generally significantly associated with richer, more diverse microbial communities than LMA sponges. Similar findings were previously reported based on different culture-independent techniques of community analysis, such as denaturing gradient gel electrophoresis (DGGE; Weisz et al., 2007; Björk et al., 2013), terminal restriction fragment length polymorphism (T-RFLP; Erwin et al., 2015), 16S rRNA gene cloning and Sanger sequencing (Giles et al., 2013), 454 pyrosequencing (Bayer et al., 2014; Moitinho-Silva et al., 2014), and Illumina sequencing (Easson and Thacker, 2014). However, it should be noted that some studies have found exceptions to this pattern (Blanquer et al., 2013; Easson and Thacker, 2014). Our data supports the increasing body of evidence showing that HMA sponges are associated with more diverse microbial communities than LMA sponges.

Our analysis further shows that microbial communities associated with HMA sponges are not only structurally distinct from those of LMA sponges, but also display smaller variation within their microbiomes than their LMA counterparts. The strongest driving force for the observed patterns was host identity, which explained the largest portion of the structural variation of microbiomes, while a smaller effect was due to the HMA-LMA status, the interaction of the HMA-LMA status and geographical region, and region alone (Table 1). These results extend previous studies that have shown the general effects of the HMA-LMA dichotomy (e.g., Bayer et al., 2014; Erwin et al., 2015), host identity (Hardoim et al., 2012; Pita et al., 2013; Easson and Thacker, 2014; Reveillaud et al., 2014; Steinert et al., 2016; Thomas et al., 2016), and geographic region (Burgsdorf et al., 2014; Luter et al., 2015) on sponge microbiomes. For the first time, these factors were ranked (Table 1). Furthermore, as demonstrated for the HMA-LMA dichotomy, we have shown that such effects are observed at different taxonomic scales, e.g., when OTU abundances are grouped at the phylum, class, or order level.

Moitinho-Silva et al. (2014) proposed that the aspect of host specificity is most appropriately addressed when considering the different OTU abundances in sponges. Subsequently, Bayer et al. (2014) introduced the term “indicator species” for certain phyla, i.e., *Chloroflexi, Poribacteria*, and *Actinobacteria* that were overrepresented in HMA over LMA sponges. In the present study, we confirm that even more taxa are differentially abundant in HMA and LMA sponges. Our analysis identifies additional phyla (e.g., *Acidobacteria*, PAUC34f, *Gemmatimonadetes*), classes (e.g., SAR202, *Anaerolineae, Acidimicrobiia*), and OTUs (e.g., Otu0000007, Otu0000004, Otu0000008) that are more abundant in HMA than LMA sponges and can thus be considered as “HMA indicators” (Figure 4). For the LMA sponges, we confirm the previously reported enrichment of *Proteobacteria* (Blanquer et al., 2013; Giles et al., 2013) and identify additional clades at the phylum (e.g., *Bacteroidetes, Planctomycetes, Firmicutes*), class (e.g., *Alphaproteobacteria, Betaproteobacteria, Flavobacteriia*) and OTU (e.g., Otu0000168, Otu0000002, Otu0000094) levels that can now also be considered “LMA indicators” (Figure 4).

### Prediction of the HMA-LMA status by machine learning

The high classification performance (>98% correctly classified samples) of the Random Forest algorithm trained with phylum and class abundances suggests that these taxonomic levels are good proxies to resolve the HMA-LMA dichotomy in sponges. Several predictions of the HMA-LMA status made by Random Forest were in agreement with previous studies. For instance, the predicted HMA sponges *Geodia barretti* and *Rhabdastrella globostellata* were previously described to harbor the HMA-indicator phyla *Poribacteria* and *Chloroflexi* (Radax et al., 2012; Steinert et al., 2016). Similarly, a dense microbial population was visualized by TEM in the mesohyl of the predicted HMA sponge *Stelletta maori* (Schmitt et al., 2011). Moreover, the microbiome of the predicted LMA sponges *Ianthella basta* and *Stylissa massa* were dominated by the LMA-indicator phylum *Proteobacteria* (Luter et al., 2010; de Voogd et al., 2015). Together with these results, further support to the predictions made by the Random Forest is provided by the co-localization of classified and predicted samples in NMDS plots (Figure 7). Why the classifiers showed less performance when trained on OTU abundances rather than phylum and class datasets remains unclear. It is conceivable that OTU abundances are less informative due to the large number of low abundance OTUs. OTUs that were more abundant in either the HMA or LMA groups may not be relevant for the classification by machine learning, since they were not necessarily present or evenly distributed in all species within the group in which they were more abundant (e.g., Otu0000094). In conclusion, the machine learning results suggested that the HMA-LMA dichotomy is a general pattern best resolved at phylum and class levels.

Our machine learning predictions on sponge species, whose HMA-LMA status was previously unknown, supports the hypothesis that the HMA-LMA dichotomy is a continuum with a highly bimodal distribution (Figure 7; Gloeckner et al., 2014). Altogether, 118 of the 135 species were consistently predicted either HMA or LMA sponges, forming the two large clusters 1 and 4 (Figure 6). Species that fell into clusters 2 and 3 behaved atypically with respect to the HMA-LMA dichotomy. Altogether, 90% of all species included in this study were either classified as HMA or LMA by TEM (Figure 1 and Gloeckner et al., 2014) or predicted by machine learning as HMA or LMA (Figure 6), and the remaining 10% were inconsistently classified. Considering the large number of species included in this study (*n* = 135), we posit that this pattern is representative of the HMA-LMA dichotomy in the natural environment. Since most collection efforts have so far explored tropical and temperate regions, further efforts should be directed to explore the HMA-LMA dichotomy in other marine environments, such as the deep-sea or polar waters. With respect to the species included in this study, their predicted HMA-LMA status provides *a priori* information on their microbiome structure, thus providing a basis for the selection of the appropriate sponges for future investigations related to their microbiomes.

Most sponge genera were composed of either the HMA or LMA phenotype. For example, the genera *Agelas, Aplysina*, and *Ircinia* contained only HMA sponge species, while the genera *Axinella, Cliona*, and *Suberites* were exclusively LMA sponges. Exceptionally, our analysis indicated some genera containing both HMA and LMA species. For instance, *Xestospongia bocatorensis* was predicted as LMA, while *Xestospongia testudinaria* and *Xestospongia muta* were characterized HMA species (Gloeckner et al., 2014). Similarly, *Cinachyrella alloclada* was predicted as HMA, while *Cinachyrella levantinensis* and *Cinachyrella* sp. were predicted as LMA sponges. In addition, the *Haliclona* spp. were predicted as LMA, although TEM observations indicated *Haliclona sarai* as HMA (Marra et al., unpubl. data). The inference of the evolutionary history of the HMA-LMA dichotomy from our results is limited by the occurrence of polyphyletic clades in *Porifera*, including the genera *Xestospongia* and *Haliclona* (Redmond et al., 2011, 2013). Therefore, future investigations focusing on the phylogenetic and evolutionary aspects of the HMA-LMA dichotomy are recommended.

## Conclusion

The Sponge Microbiome Project was queried to explore the HMA-LMA dichotomy in the largest currently available dataset on sponge microbiomes. Our results strongly support previous findings that showed a higher diversity and different microbial community structures in HMA compared to LMA sponges. A number of clades (phyla, classes, OTUs) that may be considered as HMA or LMA indicators were identified for future explorations of so far uncharacterized sponge species. Machine learning algorithms were trained on microbial community data to recognize and “learn” the HMA-LMA dichotomy. The performance of the Random Forest algorithm trained with phylum and class abundances showed the excellent predictive value of these taxonomic levels with regard to the HMA-LMA status. Consequently, Random Forest predicted the HMA-LMA status for 118 of 135 uncharacterized sponge species with high confidence. This study demonstrated the usefulness of machine learning tools to address biological questions related to host-associated microbial communities.

## Data accessibility

All amplicon data and metadata are public at the European Nucleotide Archive (accession number: ERP020690). Quality-filtered, demultiplexed fastq files are available at http://qiita.microbio.me (Study ID: 10793). OTU abundance matrix and OTU taxonomic information is available in Moitinho-Silva et al. (in review).

## Author contributions

LM and UH designed the study. GM, SL, and NW collected samples. YW, GM, SL, and NW performed microscopic research. LM, GS, SN, CH, RM, and TT performed bioinformatics analysis. LM and UH wrote the manuscript. All authors contributed to the writing of the manuscript.

### Conflict of interest statement

The authors declare that the research was conducted in the absence of any commercial or financial relationships that could be construed as a potential conflict of interest.
