# Supplementary material for: Predicting the HMA-LMA Status in Marine Sponges by Machine Learning
Source: Front Microbiol. 2017 May 8;8:752. doi: 10.3389/fmicb.2017.00752 (PMC5421222; doi:10.3389/fmicb.2017.00752)
Supplement: Supplementary file 4 [file DataSheet1.docx]

Supplementary Material

**Predicting the HMA-LMA status in marine sponges by machine learning**

**Lucas Moitinho-Silva^*^, Georg Steinert, Shaun Nielsen, Cristiane C. P. Hardoim, Yu-Chen Wu, Grace P. McCormack, Susanna López-Legentil, Roman Marchant, Nicole Webster, Torsten Thomas, and Ute Hentschel***

**Correspondence:**

*Lucas Moitinho-Silva, email: lucmoitinho@gmail.com

*Ute Hentschel, email: uhentschel@geomar.de

**Supplementary Figures**


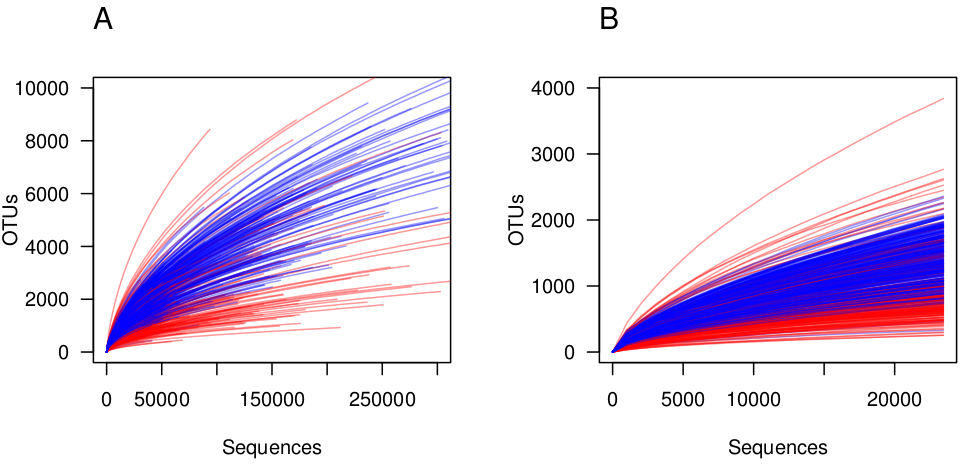


Supplementary Figure 1. Rarefaction curves of microbial communities in samples from HMA and LMA species. **(A)** Rarefaction curves were constructed based on OTU abundances in HMA (blue) and LMA (red) sponge samples. Additionally, **(B)** rarefaction curves were constructed with the OTU abundance matrix rarefied to 23,455 sequences per sample. Note that the graphs have different axes scales.


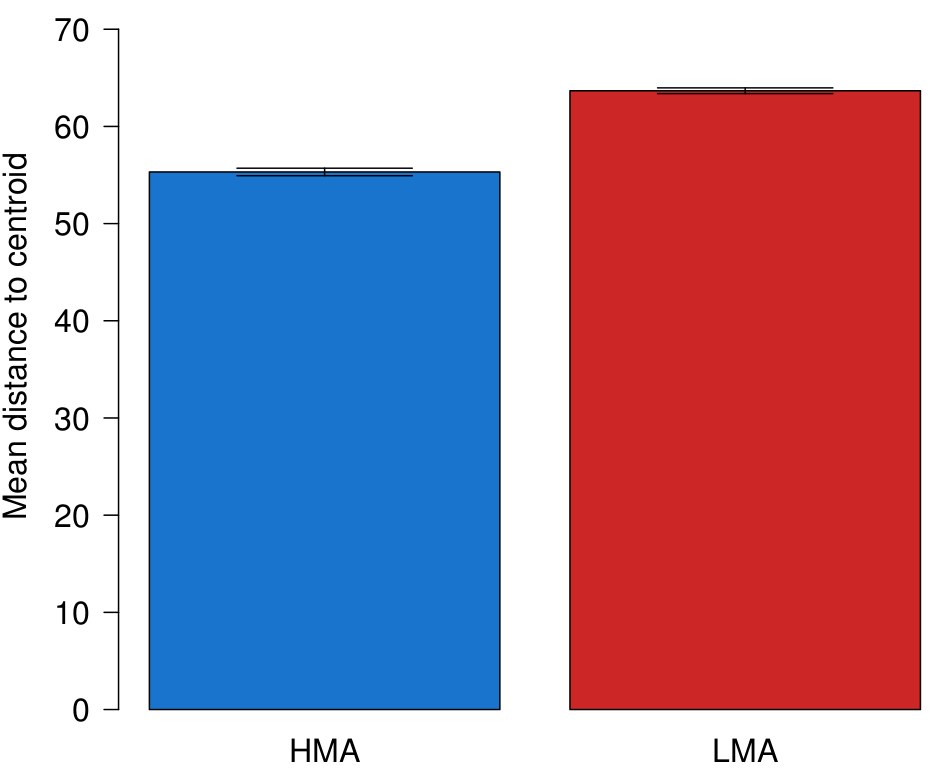


Supplementary Figure 2. Difference in dispersion between HMA and LMA sponge samples. Mean distance to group (HMA or LMA) centroid was calculated and tested with PERMDISP based on the OTU abundance matrix. P-value (t=14.906; P= 0.001) was calculated for 999 permutations.


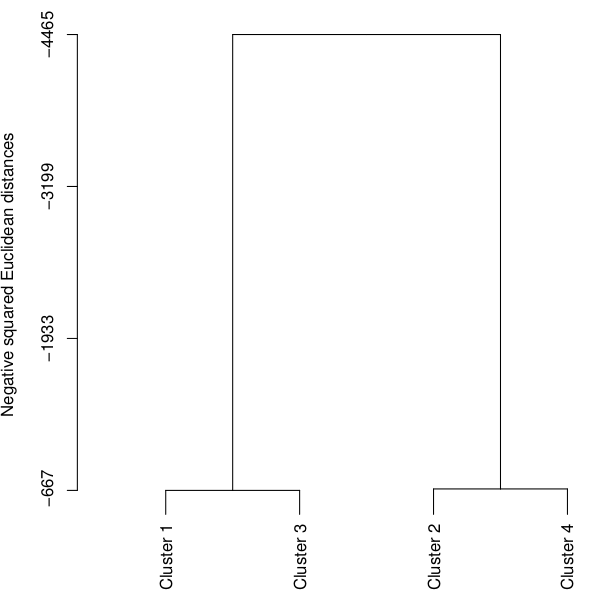


Supplementary Figure 3. Agglomerative clustering of affinity propagation clusters. Negative squared Euclidean distances were used to measure pairwise similarity between Random Forest prediction results as well as affinity propagation clusters.

**Supplementary Tables**

Supplementary Table 1. Sponge species of known HMA or LMA status.

| **Status** | **Species** | **Geographic region** | **Number of samples** |
| --- | --- | --- | --- |
| HMA | *Agelas dispar* | Caribbean Sea | 3 |
| HMA | *Aiolochroia crassa* | Caribbean Sea | 15 |
| HMA | *Aiolochroia crassa* | Southeast U.S. Continental Shelf | 2 |
| HMA | *Aplysina aerophoba* | Azores | 3 |
| HMA | *Aplysina aerophoba* | Mediterranean Sea | 18 |
| HMA | *Aplysina archeri* | Caribbean Sea | 3 |
| HMA | *Aplysina archeri* | Southeast U.S. Continental Shelf | 2 |
| HMA | *Aplysina cauliformis* | Caribbean Sea | 9 |
| HMA | *Aplysina cavernicola* | Mediterranean Sea | 10 |
| HMA | *Aplysina fistularis* | Caribbean Sea | 6 |
| HMA | *Chondrosia reniformis* | Mediterranean Sea | 26 |
| HMA | *Ectyoplasia ferox* | Caribbean Sea | 14 |
| HMA | *Erylus formosus* | Caribbean Sea | 9 |
| HMA | *Ircinia felix* | Caribbean Sea | 10 |
| HMA | *Ircinia felix* | Southeast U.S. Continental Shelf | 3 |
| HMA | *Ircinia strobilina* | Caribbean Sea | 27 |
| HMA | *Ircinia strobilina* | Southeast U.S. Continental Shelf | 3 |
| HMA | *Ircinia variabilis* | Iberian Coastal | 2 |
| HMA | *Ircinia variabilis* | Mediterranean Sea | 63 |
| HMA | *Petrosia ficiformis* | Azores | 14 |
| HMA | *Petrosia ficiformis* | Mediterranean Sea | 58 |
| HMA | *Plakortis* sp. | Caribbean Sea | 26 |
| HMA | *Rhopaloeides odorabile* | Northeast Australian Shelf/Great Barrier Reef | 8 |
| HMA | *Smenospongia aurea* | Caribbean Sea | 5 |
| HMA | *Xestospongia muta* | Caribbean Sea | 9 |
| HMA | *Xestospongia muta* | Southeast U.S. Continental Shelf | 8 |
| HMA | *Xestospongia testudinaria* | Northeast Australian Shelf/Great Barrier Reef | 6 |
| HMA | *Xestospongia testudinaria* | Red Sea | 5 |
| LMA | *Acanthella acuta* | Mediterranean Sea | 6 |
| LMA | *Amphimedon compressa* | Caribbean Sea | 17 |
| LMA | *Amphimedon compressa* | Southeast U.S. Continental Shelf | 8 |
| LMA | *Axinella polypoides* | Mediterranean Sea | 8 |
| LMA | *Chalinula molitba* | Caribbean Sea | 7 |
| LMA | *Crambe crambe* | Mediterranean Sea | 19 |
| LMA | *Dysidea avara* | Iberian Coastal | 10 |
| LMA | *Dysidea avara* | Mediterranean Sea | 13 |
| LMA | *Dysidea etheria* | Caribbean Sea | 9 |
| LMA | *Dysidea fragilis* | Azores | 1 |
| LMA | *Dysidea fragilis* | Iberian Coastal | 7 |
| LMA | *Dysidea fragilis* | Mediterranean Sea | 6 |
| LMA | *Dysidea fragilis* | North Sea | 5 |
| LMA | *Halichondria panicea* | North Sea | 10 |
| LMA | *Iotrochota birotulata* | Caribbean Sea | 11 |
| LMA | *Mycale laxissima* | Caribbean Sea | 28 |
| LMA | *Mycale laxissima* | Southeast U.S. Continental Shelf | 1 |
| LMA | *Niphates digitalis* | Caribbean Sea | 5 |
| LMA | *Niphates erecta* | Caribbean Sea | 9 |
| LMA | *Oscarella lobularis* | Mediterranean Sea | 8 |
| LMA | *Scopalina ruetzleri* | Caribbean Sea | 5 |
| LMA | *Stylissa carteri* | Red Sea | 5 |
| LMA | *Tedania ignis* | Caribbean Sea | 10 |

Supplementary Table 2. Sponge species of unknown HMA or LMA status.

| **Species** | **Number of samples** | **Species** | **Number of samples** |
| --- | --- | --- | --- |
| *Agelas cervicornis* | 3 | *Hyrtios erectus* | 3 |
| *Agelas conifera* | 4 | *Hyrtios proteus* | 5 |
| *Agelas oroides* | 11 | *Hyrtios* sp. | 7 |
| *Agelas* sp. | 4 | *Ianthella basta* | 9 |
| *Amphimedon chloros* | 3 | *Ircinia oros* | 58 |
| *Amphimedon erina* | 7 | *Ircinia* sp. | 12 |
| *Aphrocallistes beatrix* | 4 | *Latrunculia* sp. | 5 |
| *Aplysina cauliformis 'thin'* | 4 | *Leucetta* sp. | 6 |
| *Aplysina fulva* | 7 | *Leuconia* sp. | 6 |
| *Aplysina* sp. | 4 | *Lissodendoryx colombiensis* | 8 |
| *Aplysinella* sp. | 7 | *Luffariella* sp. | 3 |
| *Artemisina melana* | 3 | *Mycale grandis* | 71 |
| *Axinella corrugata* | 3 | *Mycale laevis* | 9 |
| *Axinella damicornis* | 5 | *Myxilla* sp. | 6 |
| *Axinella infundibuliformis* | 9 | *Negombata magnifica* | 3 |
| *Axinella rugosa* | 5 | *Neopetrosia proxima* | 5 |
| *Axinella* sp. | 9 | *Neopetrosia* sp. | 3 |
| *Axinella verrucosa* | 11 | *Neopetrosia subtriangularis* | 4 |
| *Axinyssa* sp. | 9 | *Pachastrella* sp. | 3 |
| *Biemna* sp. | 6 | *Paratetilla* sp. | 11 |
| *Cacospongia mollior* | 6 | *Phakellia fusca* | 4 |
| *Cacospongia scalaris* | 6 | *Phakellia ventilabrum* | 3 |
| *Callyspongia* sp. | 10 | *Phorbas fictitius* | 22 |
| *Carteriospongia foliascens* | 105 | *Phorbas tenacior* | 6 |
| *Chalinula* sp. | 4 | *Phyllospongia* sp. | 5 |
| *Chondrilla australiensis* | 3 | *Pione vastifica* | 4 |
| *Chondrilla caribensis* | 5 | *Placospongia intermedia* | 5 |
| *Chondrilla nucula* | 7 | *Plakina trilopha* | 3 |
| *Cinachyrella alloclada* | 10 | *Plakortis angulospiculatus* | 3 |
| *Cinachyrella levantinensis* | 3 | *Plakortis halichondrioides* | 11 |
| *Cinachyrella* sp. | 3 | *Plakortis simplex* | 4 |
| *Clathria* sp. | 6 | *Pseudoceratina* sp. | 6 |
| *Clathrina clathrus* | 6 | *Pseudocorticium jarrei* | 3 |
| *Clathrina coriacea* | 6 | *Ptilocaulis walpersi* | 3 |
| *Clathrina* sp. | 4 | *Raispailia hispida* | 4 |
| *Cliona celata* | 14 | *Raspaciona aculeata* | 6 |
| *Cliona celata complex* | 7 | *Rhabdastrella globostellata* | 15 |
| *Cliona delitrix* | 45 | *Rhaphoxya* sp. 2976 | 4 |
| *Cliona viridis* | 13 | *Sarcotragus fasciculatus* | 61 |
| *Coelocarteria singaporensis* | 14 | *Sarcotragus* sp. | 3 |
| *Corticium candelabrum* | 3 | *Sarcotragus spinosulus* | 13 |
| *Corticium* sp. | 5 | *Scopalina lophyropoda* | 4 |
| *Crella incrustans* | 4 | *Scopalina* sp. | 8 |
| *Crella* sp. | 8 | *Siphonodictyon (Aka) coralliphagum* | 3 |
| *Cymbastela coralliophila* | 11 | *Spheciospongia vagabunda* | 3 |
| *Dactylocalycidae* | 5 | *Spirastrella cunctatrix* | 15 |
| *Discodermia* sp. | 4 | *Spongia agaricina* | 11 |
| *Dysidea* sp. | 9 | *Stelletta maori* | 3 |
| *Erylus* sp. | 4 | *Stylissa massa* | 38 |
| *Geodia barretti* | 16 | *Stylissa* sp. | 4 |
| *Geodia* sp. | 4 | *Suberites carnosus* | 3 |
| *Halichondria phakellioides* | 4 | *Suberites clavatus* | 3 |
| *Haliclona fascigera* | 6 | *Suberites diversicolor* | 4 |
| *Haliclona fulva* | 5 | *Suberites massa* | 3 |
| *Haliclona indistincta* | 6 | *Tedania klausi* | 5 |
| *Haliclona mediterranea* | 5 | *Tedania* sp. | 9 |
| *Haliclona mucosa* | 6 | *Terpios hoshinota* | 12 |
| *Haliclona oculata* | 18 | *Tethya citrina* | 3 |
| *Haliclona* sp. | 4 | *Tethya* sp. | 6 |
| *Haliclona tubifera* | 8 | *Tetrapocillon minor* | 6 |
| *Haliclona vansoesti* | 3 | *Theonella swinhoei* | 13 |
| *Haliclona viscosa* | 3 | *Thymosia guernei* | 3 |
| *Haliclona walentinae* | 9 | *Topsentia* sp. | 4 |
| *Halisarca caerulea* | 3 | *Verongula rigida* | 5 |
| *Hemimycale columella* | 9 | *Xestospongia bocatorensis* | 7 |
| *Hippospongia* sp. | 3 | *Xestospongia proxima* | 5 |
| *Hymeniacidon perlevis* | 6 | *Xestospongia* sp. | 34 |
| *Hyrtios altum* | 27 |  |  |

Supplementary Table 3. Samples included in the study.

(in separated file)

Supplementary Table 4. Statistical comparison between alpha diversity metrics between HMA and LMA groups.

| **Measures** | **Likelihood ratio test comparison of full and null linear mixed models*** | |
| --- | --- | --- |
|  | **Χ^2^(1)** | **P-value** |
| OTU counts | 10.57 | 0.001 |
| Chao | 18.67 | < 0.001 |
| ACE | 22.41 | < 0.001 |
| Shannon | 46.24 | < 0.001 |
| InvSimpson | 53.13 | < 0.001 |
| Pielou's evenness | 48.12 | < 0.001 |
| *Models are described in the materials and methods session. | | |

Supplementary Table 5. Relative abundance of enriched phyla and classes in HMA and LMA sponges.

(in separated file)

Supplementary Table 6. Relative abundance of enriched OTUs in HMA and LMA sponges.

(in separated file)

Supplementary Table 7. Performance of classifiers trained with phylum, class, and OTU datasets.

| **Classifier** | **Phylum** | **Class** | **OTU** |
| --- | --- | --- | --- |
| AdaBoost | 94.67±11.79 | 91.11±18.52 | **91.35±19.63** |
| Decision Tree (mdt=5) | 90.73±17.03 | 89.65±20.60 | 87.86±21.59 |
| Linear Discriminant Analysis | 93.42±14.22 | 85.87±21.53 | 82.95±27.40 |
| Linear SVM (C=0.025) | 91.74±17.86 | 81.80±31.10 | 60.16±42.91 |
| Naive Bayes | 78.14±30.29 | 86.36±24.02 | 82.65±28.42 |
| Nearest Neighbors (neighbors=3) | 92.87±19.16 | 88.59±23.07 | 57.11±47.48 |
| Quadratic Discriminant Analysis | 74.20±32.07 | 77.54±26.23 | 79.08±25.93 |
| Random Forest (mdt=5, t=10, f=1) | **96.90±5.75** | **94.75±12.27** | 50.00±50.71 |
| RBF SVM (gamma=2, C=1) | 50.00±50.71 | 50.00±50.71 | 50.00±50.71 |
| Classifiers were carried out with default parameters, except when specified in brackets.  Weighted mean percentage of correctly classified samples per species ± weighted standard deviation are shown. Highest value in each column is in bold font.  Abbreviated terms are: support vector machine (SVM), Radial Basis Function (RBF), maximum depth of the tree (mdt), trees in the forest (t), and features to consider when looking for the best split (f). | | | |
